# The Genetic Variability of *APOE* in Different Human Populations and Its Implications for Longevity

**DOI:** 10.3390/genes10030222

**Published:** 2019-03-15

**Authors:** Paolo Abondio, Marco Sazzini, Paolo Garagnani, Alessio Boattini, Daniela Monti, Claudio Franceschi, Donata Luiselli, Cristina Giuliani

**Affiliations:** 1 Laboratory of Molecular Anthropology & Centre for Genome Biology, Department of Biological, Geological and Environmental Sciences, University of Bologna, 40126 Bologna, Italy; marco.sazzini2@unibo.it (M.S.); alessio.boattini2@unibo.it (A.B.); 2 Department of Experimental, Diagnostic, and Specialty Medicine (DIMES), University of Bologna, 40126 Bologna, Italy; paolo.garagnani2@unibo.it; 3 Department of Experimental and Clinical Biomedical Sciences “Mario Serio”, University of Florence, 50134 Florence, Italy; daniela.monti@unifi.it; 4 IRCCS Istituto delle Scienze Neurologiche di Bologna, 40139 Bologna, Italy; claudio.franceschi@unibo.it; 5 Department of Cultural Heritage (DBC), University of Bologna, Ravenna Campus, 48121 Ravenna, Italy; donata.luiselli@unibo.it; 6 School of Anthropology and Museum Ethnography, University of Oxford, OX2 6PE Oxford, UK

**Keywords:** apolipoprotein E, APOE, longevity, populations, genomics

## Abstract

Human longevity is a complex phenotype resulting from the combinations of context-dependent gene-environment interactions that require analysis as a dynamic process in a cohesive ecological and evolutionary framework. Genome-wide association (GWAS) and whole-genome sequencing (WGS) studies on centenarians pointed toward the inclusion of the apolipoprotein E (*APOE*) polymorphisms ε2 and ε4, as implicated in the attainment of extreme longevity, which refers to their effect in age-related Alzheimer’s disease (AD) and cardiovascular disease (CVD). In this case, the available literature on *APOE* and its involvement in longevity is described according to an anthropological and population genetics perspective. This aims to highlight the evolutionary history of this gene, how its participation in several biological pathways relates to human longevity, and which evolutionary dynamics may have shaped the distribution of *APOE* haplotypes across the globe. Its potential adaptive role will be described along with implications for the study of longevity in different human groups. This review also presents an updated overview of the worldwide distribution of *APOE* alleles based on modern day data from public databases and ancient DNA samples retrieved from literature in the attempt to understand the spatial and temporal frame in which present-day patterns of *APOE* variation evolved.

## 1. Introduction

The study of *APOE* and its isoforms has spread in all the studies about the genetics of human longevity and this is one of the first genes that emerged in candidate-gene studies and in genome-wide analysis in different human populations. The pleiotropic roles of this gene as well as the pattern of variability across different human groups provide an interesting perspective on the analysis of the evolutionary relationship between human genetics, environmental variables, and the attainment of extreme longevity as a healthy phenotype. In the present review, the following topics will be discussed.

1. *APOE* gene and protein structure and function, including the latest theoretical models describing its mechanism of action
2. The role of *APOE* in human longevity, its physiological functions, and the involvement in pathological traits in modern populations
3. *APOE* evolution and variability among human populations, including a novel analysis of modern and ancient data
4. The evolutionary mechanisms that maintained *APOE* deleterious variants in modern human populations.

## 2. *APOE* Structure and Models

Human APOE is a 299-amino acid long protein (34 kDa in weight) belonging to the family of amphiphilic exchangeable apolipo-proteins that is expressed in hepatocytes, monocytes/macrophages, adipocytes, astrocytes, and kidney cells [1,2,3,4]. Structural studies have shown two independently-folded domains for the lipid-free protein: an N-terminal elongated domain (residues 1–167) forms a 4 α-helix cluster in which non-polar residues face the inside of the protein, while the C-terminal domain (residues 206–299) has a more relaxed structure, with α-helices generating a largely exposed hydrophobic surface [5,6]. These domains are connected by an unstructured hinge that provides a large degree of mobility, which is necessary for the protein to fulfill its primary function in the hepatic and extra-hepatic uptake of plasma lipoprotein and cholesterol [7].

The N-terminal domain contains the low-density-lipoprotein receptor (LDLR) binding region, which is a cluster of basic arginine and lysine residues, spanning between positions 135 and 150 in helix 4 (an Arg-172 residue in the hinge is also necessary for the binding function [8]).A stretch of hydrophobic residues at the end of the C-terminal domain (residues 260–299) is deemed to be responsible for binding the protein to lipids as well as for directing oligomerization of lipid-free ApoE. Since the monomer is the form that binds to lipids, oligomer dissociation appears to be the rate-limiting step of protein lipidation [9,10].

The gene itself is located on chromosome 19:q13.3, together with the apoC genes *APOC1*, *APOC2,* and *APOC4*, which are members of the exchangeable lipoprotein family, and in proximity to the mitochondrial translocase of the outer membrane gene (*TOMM40*). This is another locus involved in the development of AD [11,12,13,14,15].

**Figure 1:**
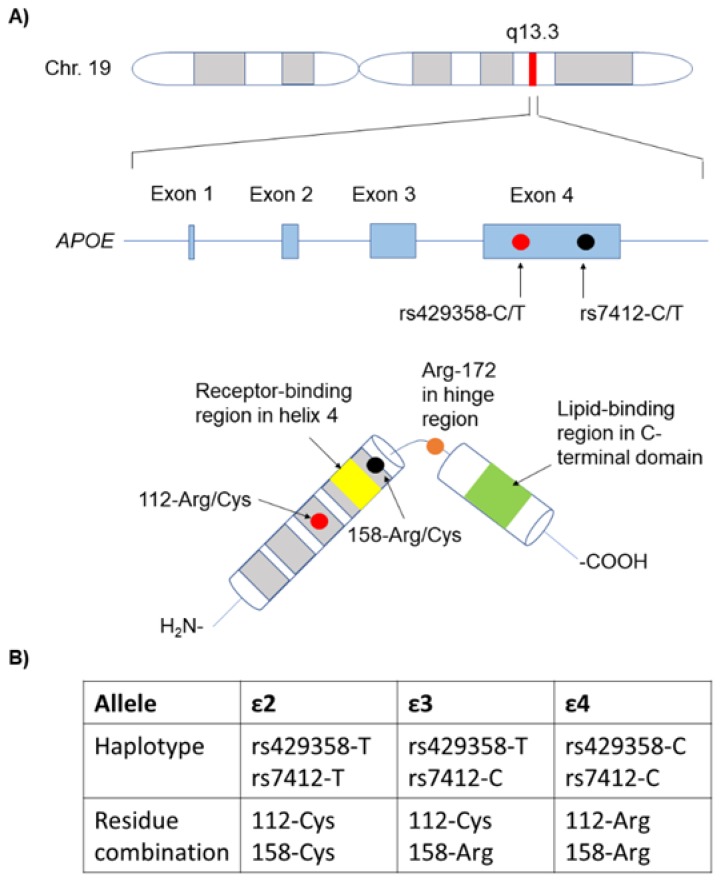
Polymorphisms underlying the three main *APOE* variants in humans. (**A**) Chromosome location, gene structure, identity of the mutating sites in the gene, and the corresponding mutating residues in the context of the protein structure. In yellow, it is indicated as the receptor-binding region in helix 4 and, in green, it is the lipid-binding region in the C-terminal domain. Red and black dots indicate the genetic variants in *APOE* and their position in the genomic and protein sequences, respectively. (**B**) Table reporting the haplotypes and corresponding residue combination associated to each *APOE* allele.

As represented in Figure 1, the combination of two mutations at the *APOE* gene (rs7412 C/T and rs429358 C/T) gives rise to the three main protein variants, called ε2, ε3, and ε4 (or, alternatively, *APOE2*, *APOE3* and *APOE4*) [16,17,18]. Isoform ε3 has a cysteine in position 112 and an arginine residue in position 158, while isoform ε2 has two cysteine residues and isoform ε4 has two arginine residues. Several other mutations can act on this background to nuance the effects of the three main variants and are involved in diverse cardiovascular pathologies, as reported, for example, in a recent review by Matsunaga and Saito [19].

While the difference in sequence is limited to a couple of residues, this has a great impact on the protein biophysical and, consequently, functional properties, since the change in structural features of APOE provides insight on the different behavior of its isoforms [20,21,22,23,24,25,26].

In particular, the Arg158Cys mutation in isoform ε2 reduces the affinity of the protein for the LDLR 50-to-100-fold [27] due to the removal of a crucial electrostatic interaction with Asp154. Mutating this residue to a neutral alanine has shown that the isoform fully recovers its functionality [28].

The mutation Cys112Arg in isoform ε4 does not change its affinity for the receptor but its preference for lipoprotein binding shifts from HDL (as do ε3 and ε2) to LDL/VLDL. This occurs because charged residues that should be buried in the protein core are, instead, propelled outwards and can establish trans-domain interactions that modify the protein structure and, therefore, lipoprotein preference, possibly by hindering overall dynamics [29,30]. Mutagenesis experiments proved effective in re-establishing the preference of isoform ε4 for HDL [17,29,31,32].

Both domain interactions and intermolecular interactions have been recently confirmed by using Forster Resonance Energy Transfer assay (FRET), which is a method to quantify the exchange of energy between two fluorescent tags attached to the ends of the APOE protein. These experiments showed that there is a consistently significant difference among isoforms, with ε4 showing a higher degree of energy transfer for both domain interaction and polymerization. However, a different study asserted that conformational changes appeared to reduce the propensity of this isoform to self-stabilize in tetramers [33,34].

Denaturation experiments aimed at testing protein stability again showed different behaviors for the three isoforms, with the ε2 N-terminal domain being the most resistant and being followed by ε3 and ε4, which is the least resistant isoform, but shows a higher number of stable intermediates between its folded and unfolded forms [35,36,37,38,39]. This has been interpreted as isoform ε4 assuming partially unfolded stable states at different pH in basic environments, facilitating large conformational changes and, in doing so, increasing the remodeling rate of lipoprotein particles. This has also been noted with other exchangeable apolipo-proteins, such as APOAI and APOAII [38,39,40,41]. Higher ε4 catabolism, although being not an index of overall increased efficiency in plasma lipoprotein clearance, may justify why *APOE4* homozygotes have a lower plasma APOE concentration [42,43,44,45]. On the other hand, it has been suggested that partially folded APOE is more sensitive to proteolysis of the domain-connecting hinge and that isoform ε4 may be more easily flagged as “misfolded” due to domain interaction, particularly in the brain [46,47,48,49,50].

It is also important to remember that no definitive mechanism for how APOE binds to lipids has been elucidated even though different hypotheses have emerged over the years, especially in relation to the implication of its isoforms in pathological traits. Starting from the concept of “molten globule” [36,51], a hairpin model has been proposed assuming that the protein bends itself so that the LDLR-binding motif is exposed at one extremity of the structure [31,52,53,54,55]. Other studies have suggested a conformational heterogeneity of bound apoE, observing that LDLR binding affinity, while higher in the bound protein than in the lipid-free protein, is modulated by the particle size, its lipid composition, and the presence of other bound lipoproteins [31,52,56,57,58].

A revised model has been recently proposed and considers the high proportion of intrinsically disordered regions in the protein (up to a third of the whole molecular structure), multiple interactions between the two domains, the presence of evolutionarily conserved residues, and structural differences that may justify the lipid-binding preferences of isoforms ε3 and ε4 [20,59]. The authors of this work also argue that most structural studies on lipid-bound apoE make use of the hepatocyte-secreted protein and plasma lipids, but that the lipid composition in the brain is different and the current models may fail to address lipidation mechanisms of astrocyte-synthesized APOE [59].

## 3. *APOE* Function and Pathology

Multiple lipid-related physiological functions are associated with *APOE*. In particular, isoform ε3 helps in maintaining the structural integrity of cholesterol-rich lipoproteins and enhances their solubilization in blood plasma, regulates lipid homeostasis of both hepatic and non-hepatic tissues, facilitates lipid internalization in cells and, when expressed by lipid-laden macrophages after cellular clearance, activates the reverse cholesterol transport, redirecting any excess of cholesterol to the liver for elimination [60,61,62,63].

The *APOE* genotype accounts for the vast majority of AD risk and AD pathology: inheriting one copy of *APOE4* raises a person’s risk of developing the disease fourfold, while, with two copies, the risk increases 12-fold [64]. Raber and colleagues and, at the same time, Saunders and colleagues reported that clinical data regarding the association of the ε4 allele with AD suggests that 50% of AD is associated with the ε4 allele in the United States [65,66]. *APOE4* may be responsible for the accelerated formation of β-pleated amyloid, as supported by studies showing that individuals with two copies of the *APOE* ε4 allele have a higher risk and earlier onset than heterozygous subject [67]. Moreover, a significant increase in risk of EOAD (early-onset Alzheimer’s disease) was found for individuals homozygous for *APOE4* regardless of family history of dementia, but an increase in EOAD risk for *APOE4* heterozygotes could only be shown in subjects with a positive family history [68].

Experiments with knock-out mice have proven that failed expression of *APOE* leads to a shortened lifespan due to the emergence of typically age-related phenotypes like an altered lipoprotein profile (the forefront of atherosclerosis and cardiovascular disease), neurological disorders, type II diabetes, deficits in immune response, and elevated markers of oxidative stress [69,70,71,72,73,74,75]. Moreover, the *APOE* variants determining the three isoforms ε2, ε3, and ε4 have also been associated with the modulation of body mass index (BMI) at statistical significance (*p* < 10^−3^) in a meta-analysis including 27,863 individuals from seven longitudinal cohort studies [76]. This highlights, on one hand, that *APOE* is a pleiotropic gene that simultaneously affects multiple phenotypes, depending on the site of protein synthesis (in particular, liver and brain). On the other hand, this emphasizes that the manifestations of its impairment fit the definition of aging as a general decline in biological functions, decreased stress resistance, and elevated susceptibility to disease that leads to an increase in mortality with age [77,78,79].

Most of the research conducted at this point focused on isoform ε4 as the “functionally altered” form of APOE in the brain since this is one of the most consistent candidates associated with human longevity and the onset of AD, according to GWAS and whole genome sequencing studies [62,66,68,80].

The finding of unexpectedly large proportions of C-terminal APOE in β-amyloid plaques of ε4/ε4 homozygous AD subjects leads to the hypothesis that the partially folded protein is highly sensitive to proteolysis [46,47,48,49,50] and this prevents APOE in helping Aβ clearance, favoring instead its deposition [81]. By folding into a more helical structure, truncated ε4-165 was shown to have deleterious effects on this same process, which stresses that structural integrity is important for AD pathogenesis [82,83,84]. The link with Aβ has also been associated with a higher degree of lysosome leakage in neurons, primarily due to the enhanced lipid remodeling activity of isoform ε4 on the lysosomal membrane at a low pH [85,86].

Experiments on mice have highlighted how isoform ε4 can also cause behavioral deficits in the absence of amyloid accumulation and, as with AD in humans, spatial and memory impairments increase with age and are observed primarily in females [87,88,89,90]. Regarding neuronal plasticity, similar studies showed that isoform ε3 associated with VLDL clearly stimulates neurite extension in developing neurons by feeding their membrane with lipids, while isoform ε4 inhibits branching likely due to effects on microtubule stability mediated by the LDLR-protein signaling pathway. The ε4 isoform also inhibits GABAergic input in newly formed neurons [91,92,93,94].

Furthermore, this isoform has been associated with decreased cerebral glucose metabolism that occurs even decades before the cognitive impairment becomes apparent, which suggests an interaction with the mitochondrial membrane and components of the respiratory complexes III and IV at very early stages of the disease [95,96,97,98,99,100]. An interesting observation is that mitochondria and the endoplasmic reticulum (ER) are intimately connected via mitochondria-associated membranes (MAMs) and the protein miofusin-2, so that mitochondrial dysfunction may propagate to the ER and affect the secretory pathway [12,101]. If the protein is recognized as unfolded, the pathways of the unfolded protein response can activate an inflammatory process by stimulating NF-kB, which is a transcription and cytokine regulator that mediates the immune response in cell survival [102,103,104].

Isoform ε4 also shows a decrease in the anti-oxidative properties of APOE as a metal cation binding protein. In fact, APOE4 genotype correlates with a higher degree of lipid oxidation and presence of hydroxyl radical levels in the blood of post-mortem patients [71,105]. Macrophages overexpressing ε4 also display membrane oxidation and generate anion radicals and, as a stress response, an increase of the anti-inflammatory protein heme oxygenase 1, was observed [106].

Moreover, it has been noted that, because of the cholesterol binding property of APOE and the fact that cholesterol is the main component of the envelope of many human-infecting viruses, the different behaviors of isoforms ε3 and ε4 may, respectively, impede or ease infections. For example, extensive work in the last 20 years showed that herpes simplex virus HSV-1 is frequently found in the brain of elderly normal patients as well as AD-affected patients, and it is thought that isoform ε4 can facilitate the process of colonization and repeated activation of latent colonies through inflammation, which exacerbates neural decay at a younger age. It is also suggested that an antiviral therapy may be effective in slowing AD progression (see comprehensive reviews in References [107,108,109]). The hepatitis C virus, on the other hand, needs APOE for assembling and the host lipid metabolism is directly involved in the viral infection [110,111,112,113,114,115]. Lastly, an interesting set of studies tried to investigate a link between *APOE* and the modulation of HIV infection as a chronic disease, now that the affected individuals can live to older ages thanks to anti-retroviral therapy. Even though the overall results are somewhat contrasting, isoform ε4 seems to correlate in different cases with the development of HIV-associated neurocognitive disorders, impaired cognition, dyslipidaemia, premature brain aging, and increased chance of debilitating opportunistic infections [116,117,118,119,120] (see also a comprehensive review in Reference [121]).

However, one of the most notable associations to be examined is between *APOE* alleles and cardiovascular disease (CVD). A study carried out on nine cohorts (eight of European and one of Chinese ancestry) of middle-aged men recruited by the World Health Organization MONICA (Monitoring of Trends and Determinants in Cardiovascular Disease) Project showed how variation in the relative frequency of the ɛ4 allele could predict 40% to 75% of the variation in coronary heart disease (CHD) fatalities among populations and how a 0.01 increase in the frequency of this allele could increase CHD death rates by 24.5/100,000 [122]. A study on follow-up data for almost 1000 Danish and Finnish heart attack survivors similarly denoted that carrying this variant can be a prognostic element, as these subjects have an 80% increased risk of dying [123]. A similar conclusion is presented by a post-mortem study, performed at the Oslo University Hospital, on over 1500 individuals who died of natural causes. In the cohort of patients presenting a cardiovascular disease (35% of the total), there were significantly more ɛ4 carriers (34% against 29%) and significantly less ɛ2 carriers (12% against 14%) than in the rest of the group (*p* < 0.05) [124]. It has also been recently recognized that, not only APOE is associated to cardiovascular risk, but also with the level of unsaturated and saturated circulating fatty acids, so that some light is being shed on how environmental and dietary factors can mediate the association between APOE variants and adverse cardiovascular events [125].

The common APOE alleles ɛ2, ɛ3, and ɛ4 are located in a CpG island and the related SNPs impact on the quantity of CpG dinucleotide, which impacts the gene DNA methylation. A recent study showed that the DNA methylation profile of this genomic region differentiates AD brain if compared to that of control subjects [126]. Moreover, a recent study on lymphocytes showed that DNA methylation in the *APOE* gene is associated with age and shaped by genetic variants in the gene [114]. A different study in African Americans also suggested that DNA methylation in blood cells may be an early indicator of individuals at risk for dementia [127].

## 4. *APOE* and Human Longevity

Many studies have attempted to grasp the complexity of the genetics of human longevity [128,129,130,131,132,133]: recent findings suggest that alleles associated with this phenotype are population-specific and, at the same time, that the achievement of extreme longevity is modulated by mechanisms shared among populations [134,135,136]. One of the most relevant loci identified by many studies (if not all) is the *APOE* gene.

Candidate gene studies, genome-wide association studies (GWAS) on geographically diverse populations, and, more recently, whole-genome sequencing approaches have been aimed at uncovering the genetic variants that influence the longevity phenotype and *APOE* possibly due to its involvement in several post-reproductive pathologies, which has emerged as a strong candidate in most of them. In this section, a brief overview of the studies on human longevity conducted in relation to the three main variants of *APOE* is presented, with special attention to its isoforms ε2, ε3, and ε4 arising from the combination of two mutations (rs7412 C/T and rs429358 C/T) [16,17,18].

Several GWAS supported the association between *APOE* and the longevity trait. For example, a Japanese study including 743 centenarians and 822 middle-aged controls found a novel positive association between variant rs16835198-G of the gene *FNDC5* (which synthetizes a pro-hormone that is upregulated by muscular exercise) and *APOE* alleles in individuals with extreme longevity, which further highlights the polygenic nature of this trait [137]. A recent meta-analysis of GWAS examined data from 6036 individuals at least 90 years old against a control group of 3757 subjects that died between the ages of 55 and 80. A replication of known variants at *APOE* and *FOXO3* genes was obtained, but the authors also pointed out the difficulty in locating new alleles associated with survival past the age of 90, possibly because of heterogeneous genetic influences combined with the fact that rare variants are not usually picked up by GWAS [80]. A novel statistical method for evaluating genome-wide associations starting from previous knowledge of age-dependent and disease-related traits that overlap with longevity (i.e., informed GWAS, or iGWAS) was applied to reduce the background SNPs possibly associated with extreme ages and to amplify potential signals that could be difficult to pick up in small centenarian cohorts [138]. Accordingly, 92 SNPs at eight independent loci (including the *APOE*/*TOMM40* locus) were found to be associated with longevity at GWAS significance (*p* < 10^−8^) and four of these were further replicated in three different validation cohorts including the *APOE*/*TOMM40* rs4420638 variant [138].

However, other studies failed to identify significant associations. For example, a study involving a Chinese cohort of 312 individuals with at least one long-lived parent (i.e., aged over 90) and 298 controls without a familial history of longevity found no significant correlation between *APOE* isoforms, age, and the levels of blood cholesterol (HDL-C) even though HDL-C levels themselves are significantly higher in the longevity group (*p* = 0.0001) [139]. The first study on a Brazilian cohort, including 220 individuals of at least 85 years of age and 232 controls averaging 72 years, was recently performed to investigate the association between *FOXO3*, *SOD2*, *SIRT1,* and *APOE* known variants and several phenotypes in oldest-olds. Only an association of two *FOXO3* alleles with gender and triglyceride levels was confirmed in this case and the authors suggested expansion of the number of samples in order to perform a more powerful analysis [140].

A similar pattern emerged from candidate gene studies, as some have highlighted putative associations between *APOE* and extreme lifespan, while others have not. For example, a study focused on three independent cohorts of centenarians from Italy, Spain, and Japan compared with healthy, younger controls confirmed the ε4 allele being negatively associated with extreme longevity in all three cases after adjustment for sex, while allele ε2 was positively associated with the same trait in the Japanese and Italian cohorts only. This highlighted that the ε4 variant appears to decrease the likelihood of reaching extreme ages across ethnicity and geographic origin [141]. A recently published paper on 450 individuals of Ashkenazi Jewish ancestry at least 95 years of age contrasted with 500 controls without a history of familial longevity, which undertook a full analysis of the coding and regulatory regions of *APOE*. Two common regulatory variants were, thus, found in the proximal promoter of the gene (rs405509 and rs769449), which is significantly depleted in the elderly group (*p* < 0.036). Moreover, a significant enrichment of the ε2 allele (*p* = 0.003) and the ε2/ε3 genotype (*p* = 0.005), as well as a reduction of the ε3/ε4 genotype (*p* = 0.005) were observed in the same group [142]. Two recent reviews and meta-analyses of polymorphisms associated with human longevity recovered genomic data of European and Asiatic cohorts involving centenarians (i.e., 13 cohorts [141,143,144,145,146,147,148,149,150,151,152,153] for the 2014 review [154], 12 cohort s [141,143,144,148,149,155,156,157,158] for the 2018 review [130]), and added newly generated data to obtain groups of at least 2700 centenarian cases and 11,000 younger controls. The first study highlighted how the likelihood of reaching extreme longevity is negatively associated with carrying the ε4 allele, the ε4/ε4, ε3/ε4, or ε2/ε4 genotypes (all *p* < 0.001), while the trait is positively associated with the ε2/ε3 genotype (*p* = 0.017) [154]. The second study ascertained the homogeneity between the European and Asian groups when accounting for ethnicity. It also confirmed a significant negative association of the ε4 allele with longevity and a positive association of the ε2 variant with the same trait (which was not supported by the 2014 meta-analysis [154]) when compared to the ε3 allele (*p* < 0.0001) [130]. In another meta-analysis, data of over 28,000 individuals born between 1880 and 1975 were collected from seven studies on population longevity and familial healthy aging, with cases ranging from 96 to 119 years and controls from 0 to 99 years. Three genetic models (i.e., standard genotypic model, additive model for the effects of the ε2 allele, grouping of genotypes containing and not containing ε4) and two definitions of longevity (i.e., age at death, age reached by less than 1% of the population) were applied. Results showed that carrying the ε2 allele, but not ε4, is associated with significantly increased odds of reaching extreme longevity, with decreased risk of death when compared to the most common genotype ε3/ε3, but modest risk reduction at the most extreme ages. The opposite is observed for ε4, which acts independently from ε2 and associates with decreased odds for extended lifespan and an increased death risk that persists even at extreme ages in all groups. Furthermore, a joint haplotype analysis of five SNPs at the *PRVL2*-*TOMM40-APOE-APOC1* gene cluster revealed that three haplotypes were individually associated with extreme lifespan when compared to the most common haplotype. The first one, containing ε2, was associated with a 34% increase in odds of extreme longevity (*p* = 7.8 × 10^−7^). The second one, containing ε4, was associated with a 50% decrease in the same odds (*p* = 10^−8^). The last one was, instead, an uncommon haplotype containing ε3 and was associated with a 20% decrease in odds for extreme longevity (*p* = 0.04), which suggests that there are SNPs at this locus that can exert a negative effect on longevity independently from the influence of the *APOE* ε4 allele [159].

A more extensive collection of GWAS and candidate gene studies performed in the last 8 years and describing *APOE* gene variants in human longevity is reported in Supplementary Table S1.

A recently published paper about genetic variants affecting viability over generations in large cohorts applied a method for testing the variability in allele frequency across different ages, after considering individual ancestry. When applied to the Genetic Epidemiology Research in Adult health and Aging (GERA) cohort and to parents of the UK Biobank participants, few common variants significantly related to mortality at specific ages were found across the genome, all tagging the *APOE* ε4 allele and the *CHRNA3* gene. When testing for viability effects of genetic variant sets, strong signals (*p* < 10^−3^) were found relating delayed puberty with longer parental lifespan, as well as later age of first birth with longer maternal lifespan and, lastly, cholesterol levels and risk of coronary artery disease, with a marked difference between male and female participants [160].

It is worth noting that recent data from Northern European populations [148,161] clarified that *APOE* variation is associated with the likelihood of reaching extreme longevity not because it is a ‘longevity gene’ that ‘ensures’ a long life by itself, but due to the fact that it is rather a ‘frailty gene’ that slightly influences mortality and, particularly, ε4 is associated with an increased risk for death that persists even beyond ages reached by less than 1% of the population [159].

## 5. *APOE* Evolution and Variability among Human Populations

Human APOE clusters with members of the groups APOA and APOC in the superfamily of exchangeable apolipoproteins. These are structurally and functionally distinct from the non-exchangeable apolipoproteins APOB48 and APOB100, which make up the core of the lipoprotein particles [162,163].

Phylogenetic reconstruction using apolipoprotein sequences from representative eukaryotic species has shown that an ancestral form of this protein already existed before Metazoan evolution (i.e., approximately 750 Mya) and that divergence between the exchangeable and non-exchangeable families is equally ancient [162]. Focusing on the human exchangeable superfamily, a similar analysis showed that APOE clusters specifically with APOA1, APOA4, and APOA5 (the most recently identified human apolipoprotein), are separated from the cluster including APOA2, APOC3, APOC2, and APOC1 (the oldest in the cluster). It is also noteworthy that the length of the synthetized protein increases from the oldest to the youngest gene [162]. When including the insect apolipo-protein ApoLpIII in the analysis, it was found to group by sequence similarity within the human APOE cluster, instead of being an outgroup to all human exchangeable proteins. This suggests that the divergence of exchangeable apolipo-proteins occurred at an early evolutionary stage, possibly with the advent of bilateral symmetry (i.e., approximately 650 Mya), while the origin of ApoLpIII has dated back to the emergence of flying insects (i.e., 500 Mya) [162,164,165]. Nevertheless, an extensive review of phylogenetic relationships among eukaryotic apolipo-proteins is not the purpose of this review [162].

Focusing on the investigation of human-specific apolipo-proteins characteristics, comparison of the protein sequence of human and primate APOE reveals that the non-human apolipo-protein has arginine in position 112, like human isoform ε4. This suggests that ε4 is the ancestral variant and recent analyses of Denisovan DNA (a specimen of archaic human found in 2010 in the Denisova cave, in the Altai Mountains in Siberia) also corroborate such a hypothesis [166,167]. Unfortunately, this information is not yet fully disentangled for the Neanderthal genomes. The other non-synonymous variants detected among the species do not alter the size or charge of the residues and are not located in functional domains [162]. The only fundamental difference, then, involves residue 61, where humans present an arginine, while all other primates have a threonine. The Thr61Arg substitution introduces a bulkier, positively charged residue near the equally charged Arg112, by which it is projected out of the N-terminal helix bundle. This repositioning allows for Arg61 in ε4 to be involved in domain interactions that affect the isoform structure, which makes the protein less stable, but readier in binding large, lipid-rich lipoproteins. It is, however, unclear how the mutation that originated human ε4 from an ancestral *APOE* could provide a net evolutionary advantage. Theories including the consumption of cholesterol-rich meat, the presence of pathogens in uncooked foods, and increasing brain size during human evolution have been proposed as well as random DNA photooxidation following the loss of body hair [162,168].

One of the most intriguing hypotheses for the development of longevity despite the presence of a deleterious *APOE* isoform, however, postulates a link with increased physical activity, over the evolutionary history of the genus *Homo*, that helped in counterbalancing a higher risk of cardiovascular disease [169]. Haplotype analysis revealed that the origin of isoforms ε2 and ε3 in humans can be dated back to 200,000 to 300,000 years ago [170], while the increase in physical exercise occurred much earlier in time, possibly around 1.8 Mya, when *Homo erectus* abandoned the sedentary lifestyle of the forests to become a hunter-gatherer. Long foraging distances and the ability to run for extended periods of time, to either follow prey or flee from danger, require endurance and increased levels of aerobic activity, which is related to the conversion of body fat into usable energy and is in stark contrast with the cardiovascular effects induced by the ε4/ε4 haplotype [171,172,173]. This likely relaxed the limitation on lifespan imposed by the deleterious allele and is in accordance with fossil dating and palaeodemographic analyses that testify an increase in the number of older individuals throughout the evolution of *H. erectus* and then *H. sapiens* [174], as well as the extension of post-reproductive lifespan in concert with the development of a hunter-gatherer lifestyle [169,175,176].

**Figure 2:**
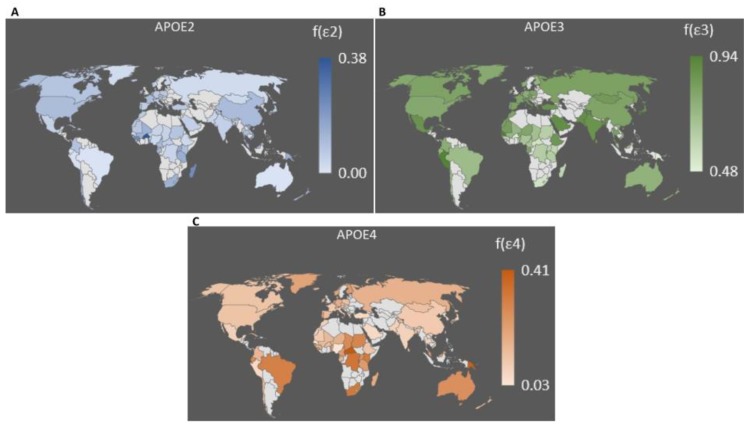
Frequency distribution of *APOE* alleles in 82 countries. Data from the 1000 Genome Project have been integrated with those published in Singh et al. 2006. (**A**) Frequency distribution of the ε2 variant. (**B**) Frequency distribution of the ε3 variant. (**C**) Frequency distribution of the ε4 variant.

However, in modern populations, isoform ε4 is only the second-most common *APOE* variant, which shows the highest frequency in indigenous populations of Central Africa (40% in Aka Pygmies, 38% in Tutsis, 33% in Zairians, and 29% in Fon), Oceania (49% in the Hui population of New Guinea, 26% in the Mowanjum aboriginal tribe of Western Australia and in Polynesians from American Samoa) and Mexico (27% for the Huychol in Nayarit [177]). Isoform ε3, instead, shows peaks of 94% in the Alberta Hutterite people of Canada, 90% in Mexican Mayas, 88% in the Basque and Sardinian populations of Europe, and 86% in Han Chinese. As highlighted in Figure 2 and reported in Supplementary Table S2, a distinct latitudinal gradient for ε4 can be observed across Europe (5% to 10% in Spain, Portugal, Italy, and Greece, up to 16% in France, Belgium, and Germany, up to 23% in the Scandinavian peninsula, with peaks of 31% in the Saami population of Finland) and it has been also reported in China (5% to 17.5% in 19 distinct populations) [178,179,180,181]. In the context of the present review, data have been also gathered for a cohort of 134 Italian centenarians and 350 healthy, younger controls, so that 484 samples were enrolled in three Italian areas (North, Center, and South Italy) and clustered according to their place of birth. DNA samples were recovered after approval by the Ethical Committee of Sant’Orsola-Malpighi University Hospital (Bologna, Italy). As shown in Supplementary Tables S3 and S4, when individuals from both groups were separately clustered by macroareas [182,183], a definite gradient could be observed for the ε4 allele in both centenarians and controls, with frequencies of 0.125 and 0.124, respectively, in Northern Italy, 0.052 and 0.063 in Central Italy, and 0.026 and 0.039 in Southern Italy. Although sample size is relatively small in the latter group, the increase in frequency from South to North at both a regional and a continental level follows a pattern that has been already observed. For example, in Italy and in Europe, for other genes involved in lipid metabolism [182,184,185], this suggests that isoform ε3 may be selected against ε4 at lower latitudes, but this does not explain the evolutionary advantage of the single amino acidic mutation Arg112Cys provided in giving rise to the now most frequent *APOE* variant worldwide [178,179,180,186]. Studies on this topic report a higher structural stability and functional flexibility of isoform ε3, which can also be associated with metal binding, oxidative stress resistance, micronutrient uptake, enhanced neuronal repair following damage, and an energy-conserving phenotype [187,188,189,190,191] (see a comprehensive review on adaptation to dietary changes in Reference [192]). However, being more adaptive and responsive to environmental changes does not justify that all the ailments of isoform ε4 is associated with, tend to be post-reproductive. Theories have been recently introduced that several derived alleles (including those at the *APOE* gene) with a protective effect on cognition after menopause may result from late-life selection through an increase in younger kin survival. The proposal of this “grandmother effect” may explain the predominance of the ε3 allele in a trans-generational way by assessing that the extension of the post-reproductive lifespan as a healthy phenotype requires the prevention of age-related cognitive decline to increase the survival of younger kin under grandparental care. Moreover, cultural transmission through generations is known to shape the social structure of modern foraging populations, which enhances the survival probability of the individuals belonging to networks that are enriched in multi-generational sharing of knowledge [175,176,193,194].

## 6. *APOE* Trade-Offs

Human longevity is a complex phenotype in which small contributions from a high number of genetic variants participate to define most age-related traits in later life. Isoform ε4 of *APOE* is involved in several cardiovascular and neural pathologies that become apparent at a post-reproductive age. Many studies in the last decade tried to find explanations as to why such a deleterious variant has been maintained at high frequency in many human groups, particularly in indigenous populations of Africa and Oceania [178]. The main collected findings suggest an association between isoform ε4 and a number of population-specific and environment-related beneficial effects that compensate for the damage induced by the same variant in later life [175,176,187,189,193].

The observation that the most detrimental effects of *APOE* (CVD, AD, reduced lifespan) mainly affect individuals of affluent populations, while most African groups do not develop significant impairment despite presenting the highest frequencies of isoform ε4, prompted a study on a rural Ghanaian population characterized by high levels of mortality from widespread infectious diseases. The analyses conducted pinpointed an association between the exposure of fertile women to high pathogen levels and a higher degree of fertility (ε4 carriers have one more child than non-carriers, while ε4 homozygous women have 3.5 more children on average). Such polymorphism may be maintained because it favors reproduction in a context where limited survival at older ages spontaneously delays the detrimental effects of the isoform. Conversely, individuals living in modernized societies, less affected by pathogens and capable of reaching an older age, have no need for the positive reproductive advantage conferred by this allele and have, instead, more probability to manifest the related negative repercussions [195]. Moreover, several candidate gene studies conducted on cohorts from industrialized countries (e.g., Iran, Turkey, United States) seem to highlight a positive relationship between cardiovascular disease and thrombophilia, as induced by lipid clearance dysfunction through the ε4 variant, and occurrences of two or more consecutive miscarriages before the 20^th^ week of gestation. These studies compared groups of affected women and fertile negative controls with at least two successful pregnancies. In all cases, a statistically significant enrichment in the ε4 variant was found for the cohorts affected by recurrent pregnancy loss as well as a significant positive association of the ε3/ε4 and ε4/ε4 genotypes with the analyzed phenotype [196,197,198,199,200].

Another study relating pathogen exposure to the preservation of the deleterious isoform was performed on the Tsimane population of Amazonian foragers. Results highlighted that ε4 carriers with a high eosinophil count (a sign of parasitic infection) perform better in cognitive tests than the non-infected carriers, irrespective of their age [201,202].

Some publications also support the thesis that the extremely long span of human survival beyond fertile age is an exception in the world of primates and mammals and is tightly linked to the practice of inter-generational cooperative child rearing, which potentially developed early in hunter-gatherer societies. The role of the grandmother is, in this case, equally parted in practices of active support, information transfer, and building of social networks that can result in extensive sharing of resources, which favor the survival and growth of the younger individuals. In this case, the positive effects of differential survival and reproductive success in early life are mirrored by deleterious cognitive deficiencies at an older age, when natural selection is absent [175,193].

Other studies have proposed that the main advantage provided by isoform ε3 when it first emerged, around 200,000 years ago, relates to an early shift in dietary habits. More organized hunting methods and the use of fire enhanced the quantity of fat-rich meat introduced with diet, which ultimately helped extend the human lifespan. Survival to reproductive age and beyond would, in this case, require both an efficient clearance of excess cholesterol from the blood and a stronger inflammatory response to food-borne pathogens, which is provided by the more ancient isoform ε4 [168,192].

The ε4 allele is an independent risk factor in age-related mortality and all-cause mortality. Since it hampers longevity, one would expect a general reduction of allele frequency with increasing age. However, the disease risk association seems to vary in an ethnic-related way. For example, hypertension and brain hemorrhage risks are increased only in Asian and European ε4 carriers [203,204], while African and Hispanic Americans show an increased risk for Alzheimer disease even in the absence of ε4, which allows for its accumulation in older age cohorts, because it is less detrimental [178,205]. Other studies have shown how this variant may exert negative pleiotropy, which grants protection to the infant brain and against infections at a younger age. This counterbalances the deleterious effects that may be induced later in life [206,207,208,209].

Lastly, isoform ε2 has a worldwide frequency of around 7% and a patchy distribution, with peaks in Southeast Asia, Australia, and some African populations (up to 19%) and absence in most indigenous American groups [178]. The effects of this isoform are opposite to those of ε4. Carriers show a lower risk and delayed onset of cognitive decline and a significantly reduced risk of cardiovascular disease, but increased infection rates at a young age [208,210,211,212,213,214]. Given the opposite effects of the two isoforms, the only current explanation for their simultaneous high frequency in several indigenous African populations is that selection acts for ε2 and ε4 against ε3, but no definitive selective mechanism has been described so far [162].

Other possible explanations for the latitudinal distribution of *APOE* variants and the maintenance of ε4 relate to its role in immunity. As highlighted by ecological and biogeographical research, there is a clear relationship between the current distribution of human infectious diseases, latitude, average temperature, humidity, and population density, with harmful bacteria flourishing in hot, wet climates and in densely populated areas of the world [215,216]. Studies involving knock-out chimeras in mice suggest that *APOE* deficiency (also mimicking reduced functionality of ε4) leads to cholesterol build-up in dendritic cell membranes, which enhances antigen presentation via lipid rafts and increasing T-cell activity (hampering macrophage function [106,217]) regardless of ensuing hypercholesterolemia. This has also been directly observed in humans, where subjects expressing the ε4 isoform have a higher activated T-cell count when compared to carriers of the other isoforms [218]. However, earlier studies on mice also highlighted that *APOE*-deficient specimens may show a significantly reduced immune response to specific pathogens by becoming more susceptible to *Lysteria monocytogenes* and *Klebsiella pneumoniae* infections [72,73,219]. As described in paragraph 3.2, several viruses require the most common form of APOE to build their particles and invade human cells. In fact, it has been observed that isoform ε4 may hamper virion synthesis and compete with the hepatitis C virus for access to LDL receptors, which reduces liver damage in exposed populations. For example, in the Italian peninsula, the North-South gradient of hepatitis C incidence overlaps with a reverse gradient in ε4 distribution [220,221]. It is less clear how the different isoforms of APOE interact with the herpes simplex virus and HIV even though the mechanisms proposed in a review by Kuhlman et al. (2010) suggest that ε4, in this case, poses less competition to cell entry, which is also helped by the enhanced presence of lipid rafts in the cell membrane [222]. A relationship between *APOE4* conservation, enhanced immune response, and pathogen distribution can be further justified by studies highlighting how carriers of this allele show higher levels of the anti-parasitic cytokine interleukin-3 (IL-3) and the pro-inflammatory tumor necrosis factor (TNFα) when exposed to endotoxins [223,224]. This seems to be especially important in the extreme case of Gram-negative infections since their toxins are membrane lipopolysaccharides (LPS) that can be collected by lipoproteins and redirected by APOE to the liver for inactivation. Reduced functionality of this protein can, thus, lead to hampered endotoxin clearance, overstimulation of macrophages, overproduction of inflammatory cytokines, and a stronger immune response leading to sepsis in the afflicted subject [225].

While local accumulation of the ε4 isoform in indigenous populations can be justified by the prevalence of infections in the absence of medical care, it can also be associated with a stronger inflammatory response to food-borne pathogens [168,192]. Other dietary factors, such as vitamin D and bone calcium assimilation, which were proven to be higher in both humans and transgenic mice carrying the ε4 allele [188,191], may have been crucial in the adaptation of populations living at extreme latitudes to the reduced amount of UV radiation. This justifies the North-South distribution of ε4 observable in Europe [188].

Many recent studies also considered a relationship between *APOE* and the gut microbiota, since, in this context, *APOE* can simultaneously exert its double role in lipid assimilation and immunity. Several experiments using *APOE* knock-out mice have shown that the diet can modulate gut microbiota composition such as with an enrichment in Firmicutes when mice were fed a typically Western diet. In turn, this relates to the amount of metabolic endotoxins in the bloodstream that stimulated a chronic inflammatory state [226,227]. On the other hand, if mice feeding on a hyperlipidic diet were immunized against their own gut microbiota, a significant decrement in serum inflammatory cytokines could be observed together with a reduction in atherosclerotic plaques, which suggests an interesting trade-off mechanism that balances the immune response against the resident microbiota with immune regulation of inflammation mediated by apolipoprotein E [228]. Other studies on obese mice and knock-out mice fed on regular chow versus a Western diet discovered that mending the loss of specific bacteria strains (e.g., *Akkermansia muciniphila*) caused by a hyperlipidic diet contrasted the enhanced permeability of the gastrointestinal tract to endotoxins and reduced vessel inflammation, fat dysmetabolism, and atherosclerosis both in normal and obese specimens [229,230,231]. Taking into consideration the immunomodulatory function of *APOE*, not only against bacteria, but also toward oxidized LDL found in sclerotic vessels, these observations highlight how both local and systemic responses can shape the overall arrangement of the intestinal biome [228].

Trade-off mechanisms may explain, in certain cases, issues regarding the replication of association signals for the same allele in different human populations and that several studies deem it more likely that a proportion of genetic influence on longevity (and of complex traits in general) may be explained through polygenic effects [232,233,234]. Furthermore, the studies performed until now did not fully address the role of rare mutations [235] nor the interaction between rare variants and *APOE* that seems to have a relevant impact on the phenotypic outcome, as supported by a recent study on the Hong Kong Chinese population [236]. Lastly, in this review, we did not address a potential limitation of trade-off mechanisms: the fact that they may be time-dependent and may be influenced by specific environmental (internal and external) conditions.

**Figure 3:**
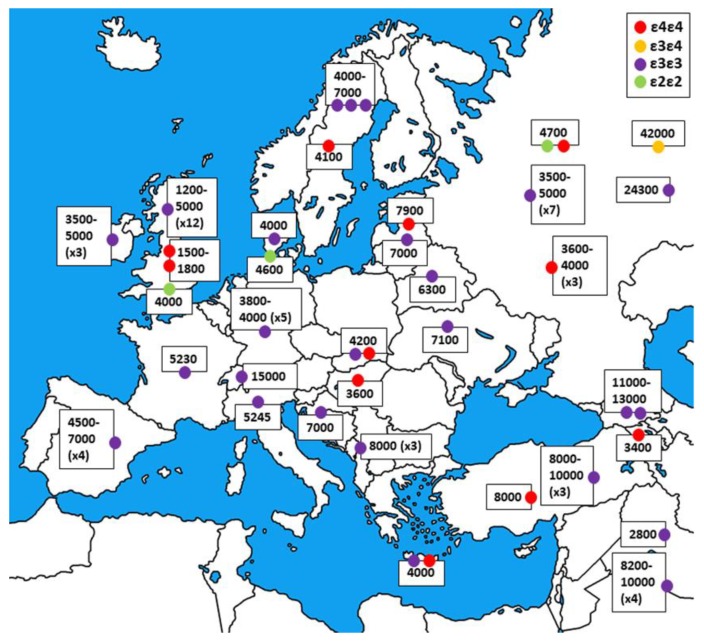
Distribution and approximate age of the analyzed ancient samples. Those coming from the same location and belonging to the same culture have been clustered together and share the same label. The number of grouped individuals is given in brackets.

The contrast between *APOE4* and *APOE3* frequency distributions in current populations, with the former being prevalent in foraging communities and the latter being predominant in regions with relevant agricultural economy, led to the theory that the ε4 variant is a relic of a hunter-gatherer genetic background that has not adapted to the modern, energy-rich, and exercise-poor lifestyle [237]. To assess the possibility of observing the temporal scale of this transition, in the context of the present review, we built a panel of 1149 publicly available ancient genomes and selected 97 of them, with both rs7412 and rs429358 already directly genotyped (the original works including the selected samples can be found at References [238,239,240,241,242,243,244,245,246,247,248,249,250,251,252,253,254,255,256,257,258]). This has been done in order to avoid the introduction of bias in the dataset by imputing variants from highly deteriorated DNA, which usually presents extended regions of missing data. The samples, mapped in Figure 3 and listed in Supplementary Table S5 with details on the place of discovery and cultural context, cover the Euro-Mediterranean area and range from 1500 to 42,000 years ago. The ε3/ε3 genotype was found to be the most frequent (83%), followed by the ε4/ε4 genotype (13%), and the ε2/ε2 genotype (3%). The only heterozygote ε3/ε4 was represented by the Ust’Ishim sample, a 42,000-year old specimen of early hunter-gatherer human found in Siberia. In more detail, the ε2/ε2 individuals are Northern European samples from the Bronze Age. Despite carrying the ancestral genotype, all ε4/ε4 individuals are less than 8000 years old, with most of them being even more recent than 5000 years, while a conspicuous number of ε3/ε3 samples are much older than this, especially in the areas of Caucasus, between the Black Sea, the Caspian Sea, and the Middle East. This temporal and spatial distribution may be coherent with Palaeolithic alleles, like *APOE4*, having been reintroduced in Europe at higher frequency with the Yamnaya migration from the Steppe during the Bronze age and *APOE3* being present at higher frequencies in the Fertile Crescent prior to the Neolithic Revolution, even though both alleles were already present in the European populations as well, as highlighted by the older local specimens [238,243,245]. However, the limited number of samples available across such an extended geographic area and the chance of genotyping errors due to the highly deteriorated ancient DNA hinder the possibility of a thorough factual discussion of the results. In order to draw more elaborate conclusions, it would be useful to recover more complete and evenly distributed ancient data, both in space and in time.

## 7. Conclusions

This review reports and summarizes relevant considerations regarding *APOE* and its pivotal role in the genetics of human longevity. Both candidate-gene studies and genome-wide analysis reveal its involvement in the attainment of an extreme lifespan by exerting a pleiotropic effect in a polygenic context. In this review, some new data (on the geographic distribution of *APOE* isoforms ε2, ε3 and ε4 in centenarians and in healthy individuals from the Italian population and on public available dataset on ancient genomes) have also been considered and evaluated in the light of the most recent findings on this gene, with particular attention to the variability across human populations. In fact, the study of the variability across different human groups is crucial to understand the differences that can be observed in the association between this gene and longevity and age-related diseases. The patterns can be justified by considering the multitude of biological pathways this gene belongs to and the different environmental conditions human populations must deal with especially with regard to pathogen exposure and dietary changes. An evolutionary perspective is also crucial to understand the conservation and current worldwide distribution of *APOE* isoforms ε2, ε3, and ε4. New data regarding DNA methylation variability in different tissues will also help more clearly define the role of this gene. Moreover, the relation between population specific cultural/ecological traits and *APOE* variability (as well as other genes) are needed to disentangle the devious way from genotype to phenotype. Given the high amount of data available on this gene, we think that an evolutionary approach, such as the one proposed by evolutionary medicine [259,260,261,262,263], will help interpret and clarify the link between even distant (or apparently not connected) results for this gene in different populations.

## Supplementary Materials

The following is available at https://www.mdpi.com/2073-4425/10/3/222/s1. Table S1: Summary of the studies published in the last eight years investigating the association between *APOE* variants and human longevity. Table S2: APOE allele frequencies in different human populations. Data used for Figure 2. Table S3: number of *APOE* alleles and haplotypes in geographically divided groups of 484 Italian centenarians and controls. Table S4: frequency of *APOE* alleles and haplotypes in geographically divided groups of 484 Italian centenarians and controls. Table S5: Summary of APOE haplotypes in ancient genomes. Data used for Figure 3.
